# Palliative Care and Heart Failure Services: Mapping National Data to Inform Policy and Service Provision

**DOI:** 10.1097/JCN.0000000000001312

**Published:** 2026-02-17

**Authors:** Ashleen Crowe, Joanne Reid, Loreena Hill, Katarzyna A. Patynowska, Tracey McConnell

**Affiliations:** 1 Ashleen Crowe, PhD, Research Fellow, School of Nursing and Midwifery, Queen’s University Belfast, Northern Ireland, United Kingdom. Joanne Reid, PhD, Professor, School of Nursing and Midwifery, Queen’s University Belfast, Northern Ireland, United Kingdom. Loreena Hill, PhD, Professor, School of Nursing and Paramedic Science, Faculty of Life & Health Sciences, Ulster University, Derry/Londonderry, United Kingdom. Katarzyna A. Patynowska, MSc, Institute of Nursing and Health Research, Ulster University Belfast, United Kingdom. Tracey McConnell, PhD, Lecturer, School of Nursing and Midwifery, Queen’s University Belfast, Northern Ireland, United Kingdom.

**Keywords:** heart failure, integration, palliative care, survey

## Abstract

**Background:**

Although palliative care is recommended for people with heart failure in various national and international guidelines, there remains no universally adopted standard on how and when to integrate it into heart failure care.

**Objective:**

To map the palliative care services available for people with heart failure in 1 country within the United Kingdom and to determine how palliative care access for people with heart failure is implemented across the country.

**Methods:**

An online survey was used to map palliative care services available for people with heart failure. Both quantitative and qualitative responses were gathered. Responses were gathered from heart failure experts, who represented heart failure service provision across the healthcare system within Northern Ireland.

**Results:**

It was found that there are gaps in integration of palliative care services into heart failure services, evidenced by 5 key findings: assessment for palliative care needs was restricted to end-of-life; no agreed systematic tool/template implemented to guide holistic assessment of unmet supportive and palliative care needs; locally agreed eligibility criteria for specialist palliative care referral not universally known; advance care plans not completed routinely, and mainly by palliative care physicians; and inequity of palliative care provision for those with heart failure with preserved ejection fraction compared with those with heart failure with reduced ejection fraction. It was also evident that the integration of palliative care into heart failure services was determined by locality.

**Conclusions:**

We identified gaps in palliative care provision for people impacted by heart failure. This enables recognition and response to these gaps to ensure delivery meets the needs of those individuals within our communities who have heart failure and palliative care needs.

**What’s New and Important**

• There is a lack of awareness amongst heart failure clinicians around locally agreed eligibility criteria for specialist palliative care referral.
• Specialist palliative care clinicians are not routinely involved in multidisciplinary meetings for those with heart failure.
• There is inequity of palliative care provision in Northern Ireland for those with heart failure with preserved ejection fraction compared with those with reduced ejection fraction.
• Advance care plans are not being completed consistently for those who have heart failure, and are mostly completed by palliative clinicians rather than heart failure clinicians.

In 2021, an estimated 64.3 million people were living with heart failure (HF) globally,^1,2^ and this number was increasing.^3–5^ The increase is due to aging populations worldwide,^3–5^ rising prevalence of underlying diseases, which are causing HF,^5^ but also advancement in treatments (such as pharmacological treatments, devices, and surgical procedures) reducing the number of sudden deaths associated with HF.^6,7^ However, as people live longer with HF, they also develop chronic physical symptoms (including shortness of breath on activity and pain), increased psychological symptoms (anxiety, depression), and social burden (in the context of HF, this is the increased limitations upon patients’ and caregivers’ daily lives).^8–11^

People with HF have comparable palliative care (PC) needs to people with cancer, yet many of these needs go unmet.^12^ Similarly, they are less likely to be referred for PC compared with those with cancer.^13,14^ The National Confidential Enquiry into Patient Outcomes and Deaths titled “Failure to Function” found that 97.7% of hospitals in England had access to PC (this was a report carried out in England and Wales highlighting the process of care for patients aged 16 years or older who died in hospital following an admission with acute HF).^15^ However, only 12.5% of those with an established HF diagnosis received PC services despite 81.3% of the deaths being anticipated upon admission to hospital.^15^

According to the World Health Organization, PC is an approach that enhances the quality of life for patients and families facing life-threatening illness by preventing and relieving suffering through early identification, assessment, and treatment of physical, psychosocial, and spiritual issues.^16^ It is of note that PC provision is not dependent on prognosis but rather should be delivered based on need from the point of diagnosis.^17^ This is especially important in the context of HF, where each individual has a unique and often complex and unpredictable illness trajectory. Therefore, the need to integrate PC may be required at different times throughout the individual’s HF journey, rather than as a one-off event. Early PC assessment and treatment would therefore be of significant value to those living with HF, and their carers, and could alleviate suffering, with the aim of improving their quality of life.^18–20^

Although PC is recommended for people with HF in various national and international guidelines^19,21–23^ there remains no universally adopted standard—in the United Kingdom, Europe,^24^ or the United States of America^25,26^—on how and when to integrate it into HF care, highlighting the need to understand current practices to inform future policy and address service gaps.

Thus, the aim of this study was to map the PC services available for people within 1 country in the United Kingdom, and to determine how PC access for people with HF is implemented across the country.

## Methods

### Design and Participants

This national study was conducted in Northern Ireland, which has an estimated population of 1.92 million, where 1 in 6 people are aged 65 and over.^27^ The study design was a regional exploratory “mapping” exercise conducted via an online survey (see Supplemental Digital Content 1, https://links.lww.com/JCN/A394).^28^ Quantitative and free-text (qualitative) data were collected via Pavlovia (a repository and launch platform for experiments, including surveys).^29^ The survey was developed from the recently commissioned (2023) National Health Service England survey. That survey was planned to map current practice across all integrated care systems to gain an understanding of the current provision of PC for people with HF in England. It was developed and carried out by a team of researchers from the University of Hull, supported by the Hull University Teaching Hospitals NHS Trust.^30^ Our survey, developed from this National Health Service England survey, was then piloted with a PC consultant and HF nurses (n = 6) to ensure it was applicable to the regional context by adapting options for organization type and professionals involved.

Eligibility criteria included:

• Health Service HF experts, with responsibility for the care of patients with HF,^31^
• Able to complete the survey,
• Willing to provide data by submitting the survey (implied informed consent).

Northern Ireland has 5 geographically distinct Health and Social Care Trusts, which are responsible for managing and delivering health and social care services. A combination of purposive and convenience sampling was carried out when selecting participants across all 5 Trusts. The sampling was purposive in that the researchers targeted expert clinicians in each HF service (differentiated by Trust) as their specific knowledge and expertise were deemed highly relevant to the study.^32,33^ This was supplemented by convenience sampling in that the researchers had an established relationship with a member of the research advisory group to act as a gatekeeper (a clinician who authorizes and facilitates access to resources within the health service)^34^ for recruiting HF nurses.^32^ This Research Advisory Group (n = 7) consisted of representatives from key organizations historically involved in ensuring the best possible health and social care for those with HF, including: Heart Failure Warriors NI, the British Heart Foundation, Chest Heart and Stroke, Marie Curie, and professionals working within the United Kingdom Health Service.

### Ethics

Ethical permissions were attained from the Faculty of Medicine, Health, and Life Sciences, Queen’s University Belfast, before the study commenced (reference number MHLS 24_162).

### Recruitment and Data Collection

Recruitment commenced on January 20, 2025, and closed on March 24, 2025. Eligible participants were identified through their professional networks and sent an invitation email by members of the research team (TM and AC). This included a link to the survey and an explanation of the purpose of the mapping exercise. If the respondent felt they could not answer all questions in the survey, they were asked to share the survey link with the relevant member(s) of their team who could provide a full picture of the PC provision for those within their HF service. Participants were informed that completion of the survey would be taken as implied consent. A reminder email was sent after 1 and 2 weeks. After receipt of this first round of responses, the research team (AC and TM) extended the survey invitation to more individuals involved with managing the HF services via a clinical gatekeeper. This was due to missing data and the need to provide a full data set to understand regional variation in PC provision.

Demographic data (including profession of the responder and Trust area) were collected from participants. This information was anonymized using unique identifiers for each Trust area. Survey topics included questions about services for people with HF, access to PC for people with HF, multidisciplinary team meetings, and PC training or teaching for clinicians (see Supplemental Digital Content 1, https://links.lww.com/JCN/A394).

### Data Analysis

Data analysis was conducted by AC, and demographic information from the survey was aggregated and summarized by AC. The quantitative survey data underwent descriptive statistical analysis. This was to illustrate PC service provision for individuals with HF. Free-text qualitative responses were subjected to codebook thematic analysis, which involved initially coding the data, then using a codebook to help organize the coding process, and then developing themes and patterns.^35^ The qualitative data were mapped to quantitative findings to provide further explanation of PC service provision.

### Rigor

The combined convenience and purposive sampling strategy used in this study meant that people with the required depth and breadth of expertise were included to provide important knowledge of the issues under investigation (purposive sampling).^36^ Data triangulation, whereby multiple perspectives on the qualitative data within the survey allowed verification of the free-text response data.^37^

## Results

As stated above, each of the 5 trusts within the regional health service has 1 HF service. However, within a Trust there can be multiple clinical sites with their own HF clinics and staff, and thus variation in services within and across trusts. This resulted in a variety within the responses from staff members (n = 10 survey responses). Qualitative and quantitative responses are presented together throughout.

Responses were received from all 5 Trusts across the region. The professions of those who responded to the survey included medical doctors and registered nurses. Respondents from each Trust stated that the clinical lead in each Trust HF service was a cardiologist with an HF interest. All respondents bar one (community) were from a hospital service.

### Identification of Patients With Unmet Supportive and Palliative Care Needs

Four out of the 5 Trusts reported use of a PC holistic needs assessment tool. However, use of a holistic assessment tool was not reported by all respondents within the same Trust, showing that there is variation within each Trust in how patients with PC needs are identified.

There was also variation within and between Trusts in relation to whether holistic assessment was carried out routinely or only occasionally. For example, although one respondent had already stated that holistic assessment was occasionally completed by HF nurses or advanced nurse practitioners, the following qualitative response suggested that identification of PC needs is “sporadic” and restricted to the end-of-life stage.

“Commonest mode would be during periods of acute deterioration as an in-patient at the end of life… mostly ad-hoc in clinical appointments - by their nature these are sporadic.”

One Trust, which reported no completion of a holistic assessment, did report using “general criteria” to identify patients with HF with unmet supportive and PC needs. These general criteria included being unable to take oral fluids/medication, increasing unresponsiveness, increasing weakness, and being bedbound. They also specified that criteria for advanced HF were used as part of holistic assessment of unmet supportive and PC needs, including:

“No further intervention likely to help. Deteriorating renal function. Hyponatraemia/anaemia. Hypotension/persistent tachy at rest. Exudation from lower limbs. Life threatening comorbidities.”

There was also wide variation in relation to awareness around the use of a systematic tool/template to guide holistic assessment of unmet supportive and PC needs. Only one respondent out of 4 across all Trusts was aware of such a tool being used.

“NISAT [Northern Ireland Single Assessment Tool] was regionally introduced across a number of years and this is what was always promoted [for] holistic assessment (certainly across community nursing). However, it wasn’t favoured across nursing [and so it] went back to a nursing assessment format. We are very aware of IPOS [Integrated Palliative care outcomes measures] and we are trying to see how it could be implemented within SPC [Specialist palliative care] services.”

In relation to who carries out a holistic assessment of unmet supportive and PC needs (using a systematic tool or not), all respondents apart from those in 1 Trust reported a range of staff, including cardiologists and nursing staff, who shared responsibility for this assessment (see Figure 1 or Supplemental Digital Content 2, https://links.lww.com/JCN/A395).

**FIGURE 1.**
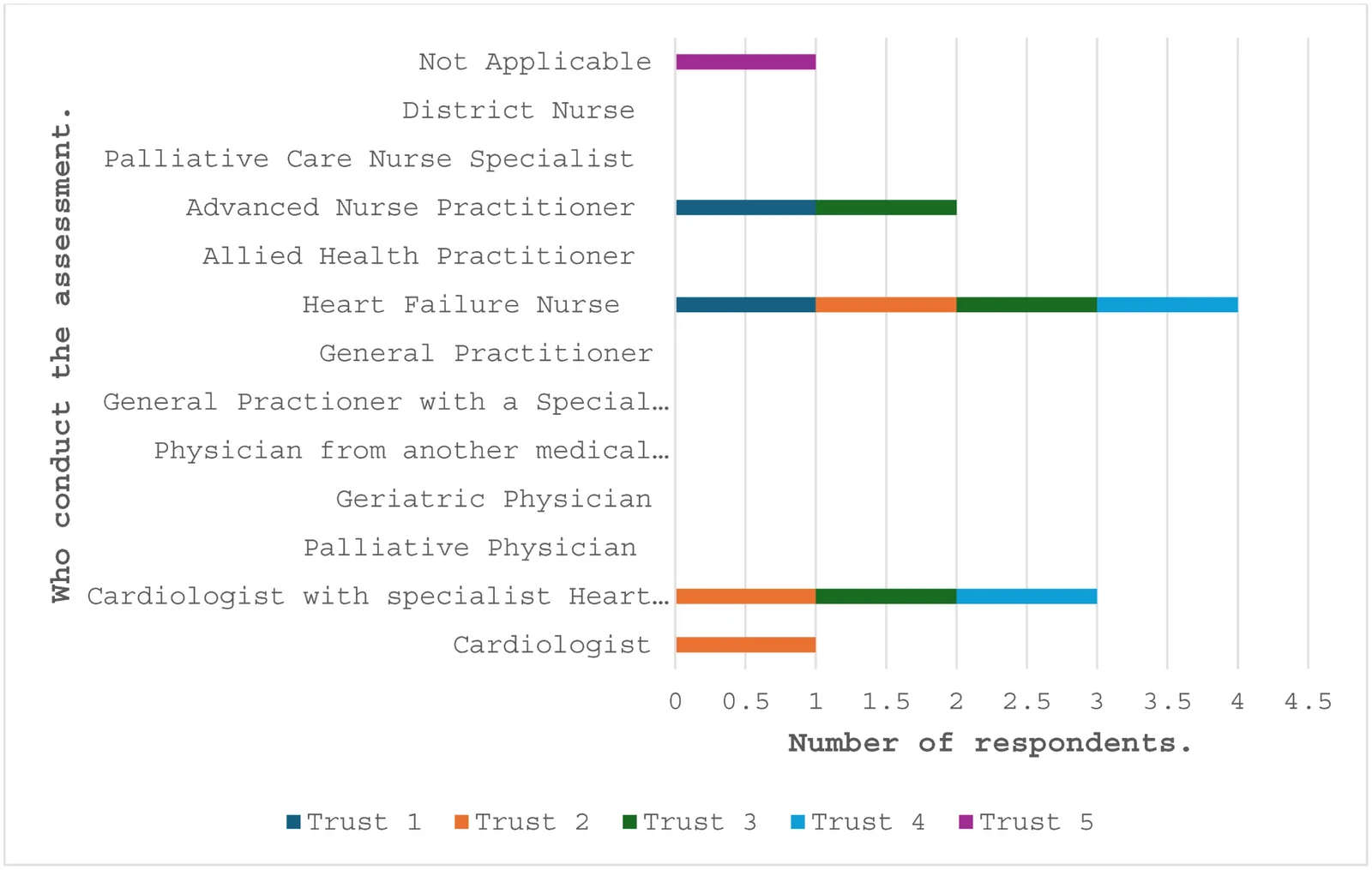
Responses to the Question “Who Conducts the Assessment Identifying Those With Unmet Supportive and Palliative Care Needs?”.

Participant responses highlighted differences that currently exist in PC provision between people with HF with reduced ejection fraction (HFrEF) and those with HF with preserved ejection fraction (HFpEF). For example, respondents from 2 Trusts reported inequities in the system for assessment of unmet PC needs for those with HFpEF.

“HFpEF not routinely under review in our HF service.”

“[There is] no funding in service [for HF nurses] to see HFpEF... Only seen by cardiologists.”

Referral to specialist PC services

Respondents from all 5 Trusts reported making referrals to specialist PC services (for whom PC is their core business), with the hospital PC team being the only service all 5 Trusts reported knowing they have access to (see Figure 2). A smaller number of Trusts reported having access to a Community PC clinical nurse specialist; Specialist palliative medicine outpatient clinic, Hospice at Home, or Trust allied healthcare professional PC (see Figure 2).

**FIGURE 2.**
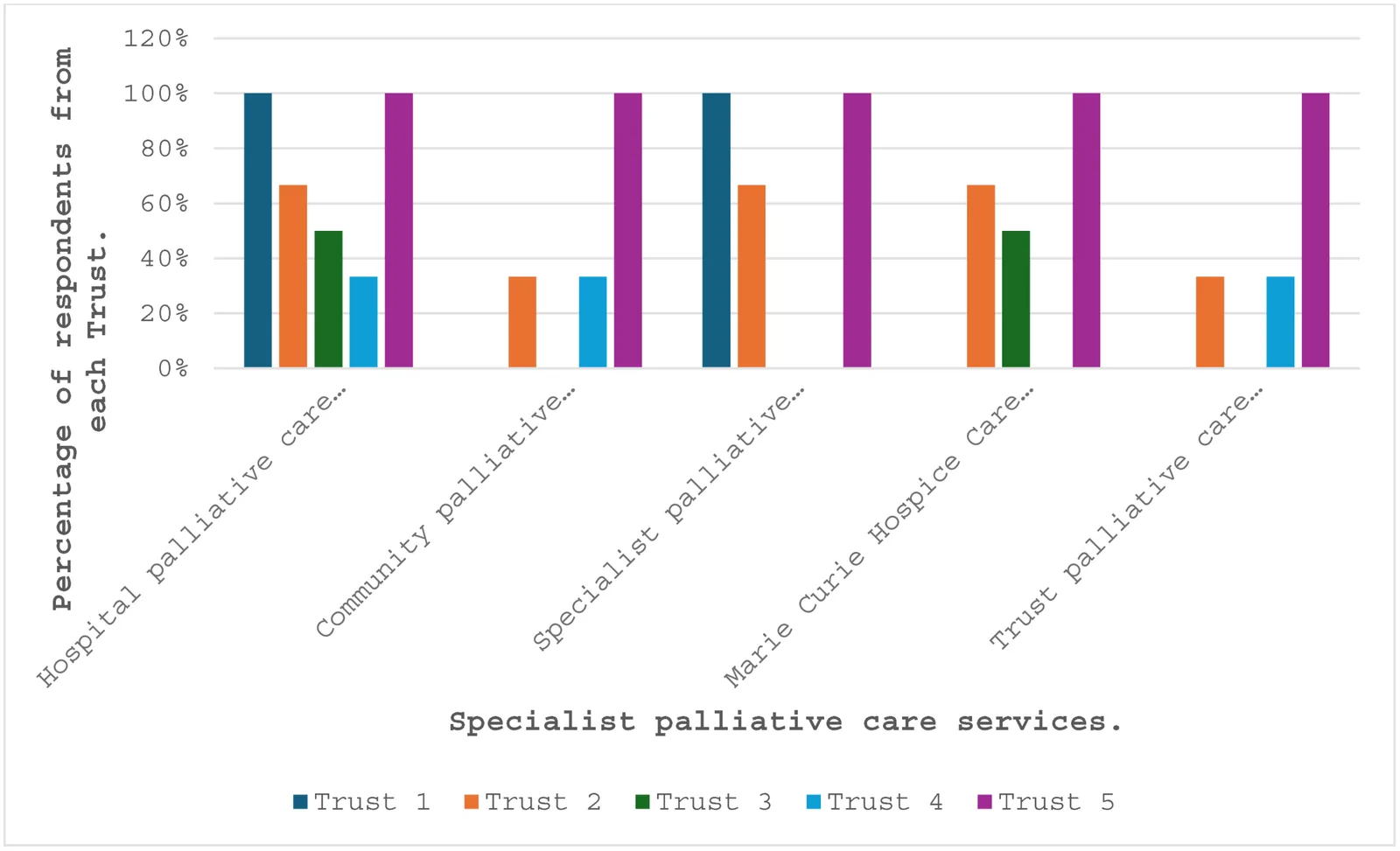
Responses to the Question “What Specialist Palliative Care Services Do You Have Access to?”.

Although all 5 Trusts reported referring to specialist PC services, there was variation within and across Trusts when reporting on access to a specialist palliative medicine consultant. Although the majority of Trusts had at least 1 respondent stating that they do have access to a specialist palliative medicine consultant, not all respondents within the Trusts stated this consistently. Similarly, there was uncertainty across the Trust Respondents in relation to having locally agreed eligibility criteria for specialist PC referral. Although 3 respondents from 3 Trusts reported having locally agreed eligibility criteria, other respondents from the same Trusts reported that they were not aware of these criteria, or that their Trust did not have such criteria (see Figure 3).

**FIGURE 3.**
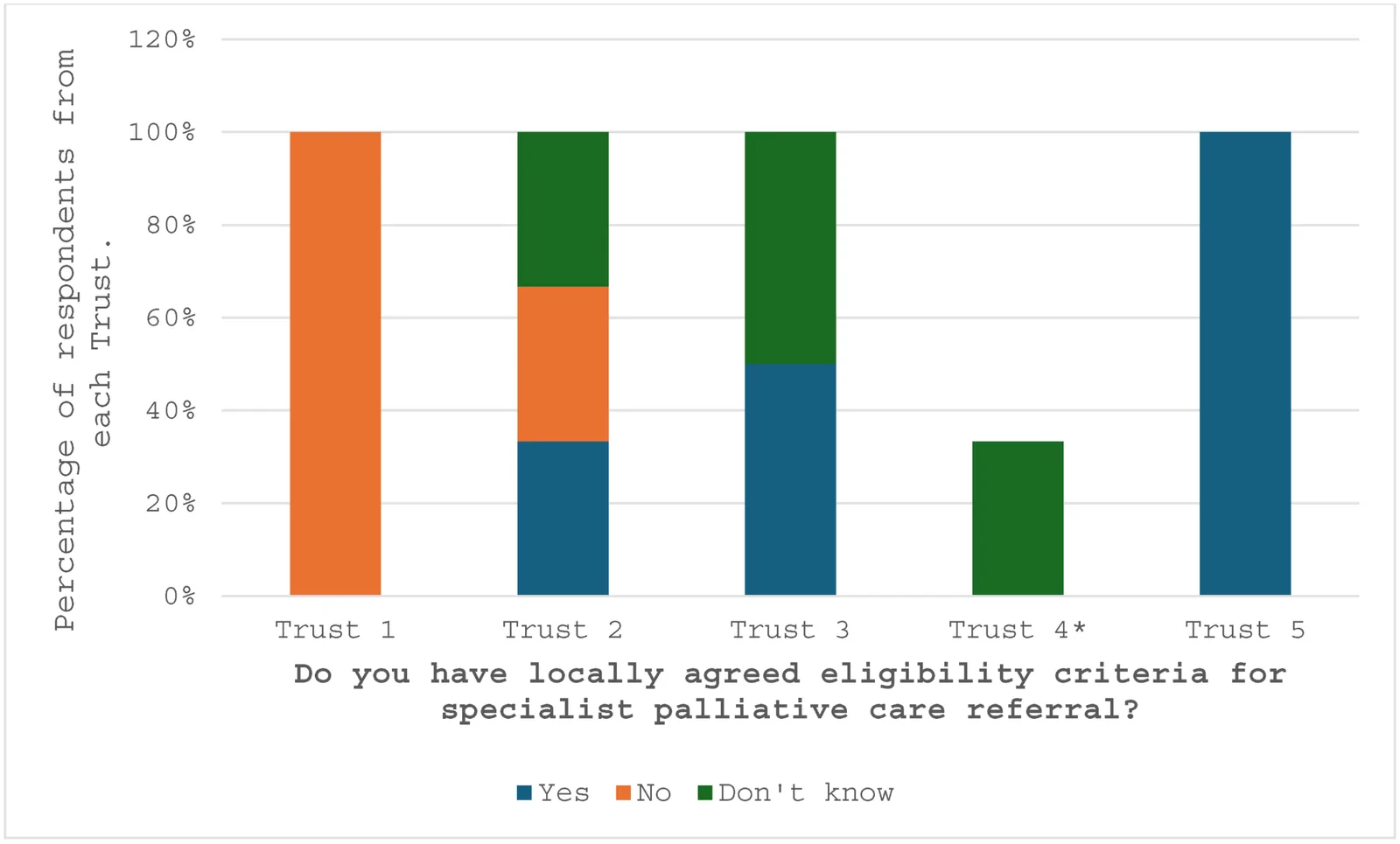
Responses to the Question “Do You Have Locally Agreed Eligibility Criteria for Specialist Palliative Care Referral?”. *Please note 2 of 3 respondents from Trust 4 did not refer to palliative care services and so were excluded from answering this question.

Two Trusts reported having the same range of locally agreed eligibility criteria for specialist PC referral, including: difficult to manage symptoms; New York Heart Association III/IV; expected to die in the next year. Only 1 Trust included further eligibility criteria of: no further cardiac treatment possible.

### Completion of Advance Care Plans

Completion of advance care plans (ACPs) varied across the region. Advance care plans can include ceiling of care plans,^38^ which outline what level of medical intervention a person wishes to have at the end-of-life (from a focus on comfort and relief of symptoms, to the highest ceiling of care, eg, all possible medical interventions), or treatment escalation plans which outline personalized care goals and preferences if the person becomes critically ill. At least 1 respondent from 4 of the 5 Trusts reported completion of ACPs. However, completion of ACPs was not consistently reported within Trusts, where in 2 Trusts there were respondents who said their service does not include completion of ACPs and others who said their service does.

Respondents from 4 Trusts reported that completion of ACPs was not routine practice. Qualitative responses showed that if completed, this was:

“Generally at physician/nurses discretion in response to deterioration/frail patient.”

It was also reported that it was most often palliative healthcare professionals who completed ACPs. One Trust stood out in having the most variety of professionals reported as completing the ACPs alongside palliative healthcare professionals (including a general practitioner with special interest, advanced nurse practitioner, PC nurse specialist, and district nurses) (Supplemental Digital Content 2, https://links.lww.com/JCN/A395).

Access to the patient’s ACP varied across the region, where only 2 Trusts reported the patient having a copy. Respondents from 3 Trusts said all hospital staff had access. Only 1 Trust reported that general practitioners and the ambulance service having access to the ACP.

In response to whether there is a systematic tool/template used to guide this assessment (for example: ACPs), answers varied between yes, no, and don’t know. The 2 respondents who said their service does have a systematic tool/template for the ACP elaborated that:

“There is a basic template but [it] asks re preferred place of care. CPR/DNR instructions. ICD deactivation & removal for cremation.”

“This is a difficult one. We do have our Advance Care Planning Policy for the Trust, which has adopted the new regional policy. As you will know the regional work on ACP has been stalled and full implementation did not get completed. We do have the regional ACP booklet (‘Your Life, Your Choices’) for the public with the templates in the back for people to complete. So people can hold their own ACP but [their ACP] will not be on Encompass [platform for single digital care records rolled out across Northern Ireland in 2025, replacing paper care plans].”

### Heart Failure Multidisciplinary Team Meetings

When asked if the HF service in their Trust has a HF Multidisciplinary Team meeting, the majority of Trusts said they do. Only 1 Trust stated that a specialist PC clinician attends, and that they attend only sometimes. In relation to communicating plans to address unmet supportive and PC needs with primary care, 3 Trusts reported using multiple methods, including electronic care records, via PC nurse specialist, via HF nurse specialist, and by letter/email. In contrast, 1 Trust reported using only 1 method of communication with primary care to address unmet supportive and PC needs (via the HF Nurse Specialist) (Supplemental Digital Content 2, https://links.lww.com/JCN/A395).

### Palliative Care and Heart Failure Training

Of the PC training options available for HF clinicians, responses showed that clinicians from all the trusts had engaged in at least one of the following: Princess Alice Essential Certificate in PC; placement with a specialist PC team (hospice or hospital); or PC in-person teaching facilitated by the PC team. One respondent elaborated that it was HF nurses from their Trust who availed of the placement with a specialist PC team, and another respondent elaborated that some clinicians from their service had completed a PC module, in addition to the other possible training options listed above (Supplemental Digital Content 2, https://links.lww.com/JCN/A395).

Similarly, HF teaching for PC clinicians appears to vary across the region (60% respondents said HF teaching was provided, 40% said it was not). Qualitative responses provided more insight into the provision of HF teaching to PC clinicians.

“[We have] Joint teaching with heart failure specialist nurses and PC specialist nurses.”

“We have met with the PC Consultant from the [hospice] and PC specialist nurses last year and plan further collaborations. Our community HF Team also link in with her.”

### Perspectives of Heart Failure Clinicians on Specific Palliative Care Components and Current Business Plans for Service Improvement

The majority of the Trusts considered every element to be very important, however, there were (see Supplemental Digital Content 3, https://links.lww.com/JCN/A396), different levels of importance in specific PC components. For example, “patient identification” and “training/education in palliative care for heart failure clinicians” were rated on average as the most important, with “anticipatory care planning” and “access to specialist palliative care” being rated on average as the least important (though both still with 70% of total respondents deeming it very important). Only 2 Trusts reported having palliative and end-of-life care included in their current service specifications and business plans for service improvement.

The results of this study highlight the gaps in integration of PC into HF services and help us to understand how PC access for people with HF is implemented across the country (Figure 4).

**FIGURE 4.**
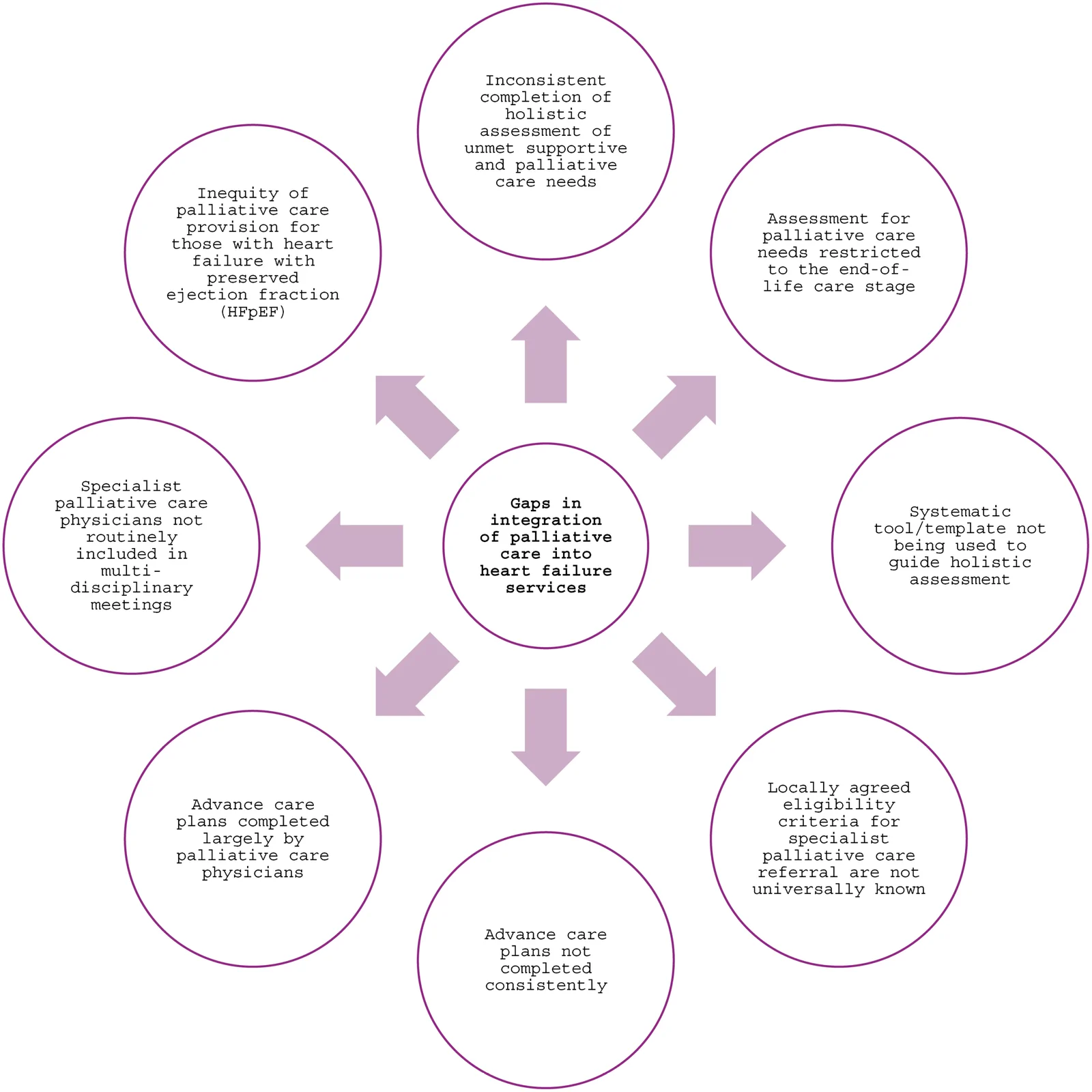
Overall Summary of Gaps in the Integration of Palliative Care Into Heart Failure Services.

## Discussion

Current recommendations (published in 2020) for integration of PC into HF services include: consider PC needs throughout the HF trajectory; address preferences and needs for treatment; focus of care should be optimal symptom control; a specialist and generalist PC approach by all professions; patients, families, and caregivers’ preferences for treatment and care should be taken into account.^24^ However, we can see from the results of this survey that these recommendations are not being carried through into practice. The results from this survey provide insights into PC provision for those with HF in 1 country within the United Kingdom, and for clinicians working in hospital HF services in high-income countries. Despite existing recommendations, key findings have highlighted ongoing gaps in PC provision for people with HF (ACPs not completed consistently, unused systematic tools for referral, lack of knowledge about specialist PC referral, and inequity of care for HFpEF), and a lack of integration of PC into HF services based on locality. The results of this survey reflect international literature,^39^ which highlights the ongoing lack of integrated PC into HF services.

One key gap in PC provision is the lack of ACPs for people with HF. Timely completion of ACPs can reduce unnecessary and distressing transfers to the hospital at the end-of-life.^40,41^ ACPs can also improve well-being by facilitating multidisciplinary collaboration and comprehensive, holistic symptom management.^42,43^ Our results also show that when ACPs are completed, it is palliative clinicians who complete them. This raises the question of how, when, or what triggers cause clinicians to ask a palliative healthcare professional to complete an ACP? A systematic review by Schichtel et al^44^ concluded that advance care planning should be introduced at a significant milestone in the person with HF’s disease trajectory (after an unscheduled hospital admission, before hospital discharge, or after a deterioration in the patient’s health status) to facilitate patient engagement with advance care planning. Research suggests that trained facilitators are best suited to complete ACPs with patients who have HF,^44^ and HF clinicians can be trained to fulfill this role. PC training and education for HF clinicians are required to facilitate this. That, and embedding specialist PC healthcare professionals within multidisciplinary HF teams would have immense value to the patient in facilitating ACP completion.^44^

Our results regarding the gaps in PC provision for those with HF support those identified by research in the United States.^45^ Chuzi et al^45^ found that only 12% of respondents reported a triggered referral to PC based on use of a systematic PC needs assessment tool, with most reporting PC referral based on the discretion of the physician. Likewise, a survey conducted in Japan showed that although PC is necessary for patients with HF, it is only considered for terminal patients with HF.^46^ The internationally relevant question is then how can integration of PC into HF services be encouraged or facilitated? A realist synthesis by McConnell et al^17^ highlighted that barriers to integrating PC into HF management can be explained by healthcare professionals’ capacity, opportunity, and motivation. This means that integration of care can be hampered by misunderstandings and absence of knowledge, where clinicians can be aware of guidelines, but do not have the capacity, motivation, or opportunity to act on or implement said guidelines.^17^ For instance, an example of this is where clinicians are operating under the misconception that PC is only for end-of-life, evidenced by holistic assessment not being completed until these end stages. Another example could be the higher rate of ACP completion by PC healthcare professionals due to a lack of knowledge among HF professionals that they could be initiating PC and end-of-life care conversations. Or also where specialist PC professionals are not being routinely included in multidisciplinary meetings because HF clinicians are simply unaware of their value in supporting HF management. The challenge to integrate PC into HF services is a consistent international problem, and the specific reasons why integration is not happening are likely very specific to the given context. More research is required to determine a roadmap to successful integration of PC into HF services.

Furthermore, survey results showed a lack of locally agreed eligibility criteria for specialist PC referral. This means that eligibility criteria for referral are left largely up to the clinician’s own experience, education, judgment, and awareness, and suggests a lack of robust referral pathways within specific organizations. Categories of eligibility criteria for PC referral can include mood disturbance, dyspnea (shortness of breath), fatigue, refractory pain, and weight loss, but consensus on these eligibility criteria is currently missing in the literature.^47^ An Australian study showed that PC referral was affected by clinicians’ personal judgment, and their focus on medical management until very end stages of HF,^48^ which was also seen in our survey, with referral to PC services often being left until the end-of-life stage. Evidence-based eligibility criteria need to be prioritized and accessible for clinicians working in HF services, along with clear referral pathways to specialist PC services when appropriate.

Diversity in PC provision for those with HF was evidenced by the different approaches across Trusts in relation to whether HFrEF and HFpEF have access to PC services. It is known that there is minimal guideline-directed medical therapy for those with HPpEF,^49^ despite evidence of its effectiveness^49^ (all patients with HFpEF are now offered sodium-glucose cotransporter-2 inhibitors as per HF guidelines).^23^ This creates a barrier to equitable PC provision for all those who have HF.

Overall, the results of this survey show that there is diversity in PC provision in HF services based on locality (this can be described in the United Kingdom as a postcode lottery, where postcode, or zip code, determines the care received because of “the lottery” of what healthcare services are available in each location). Palliative care service provision appears to be based on the patient’s address and services available in that locality, rather than on their PC need, reflecting studies conducted in America and Australia.^50,51^ There is thus an international need for routine availability of PC, facilitated by integration of PC into HF services regardless of where the person lives.

There are a number of recommendations that can be drawn from the findings of this work:

• Targeted education initiatives need to be developed and implemented for clinicians and the public (including through a public health campaign) to improve understanding of what PC is, its benefits, and when it should be introduced.
• Evidence-based PC needs assessment tools, and locally agreed referral criteria for specialist PC should be embedded into electronic health records where they exist, and/or patient notes to prompt completion by HF clinicians (including at the point of diagnosis, and throughout HF treatment, not only towards “end-of-life”)
• Advance care planning should be standard practice within HF management.
• Specialist PC professionals should be part of HF multidisciplinary team meetings. Healthcare systems should ensure that meeting structures, referral processes, and communication pathways enable regular PC input.
• Develop and implement inclusive referral criteria and clinical guidance that explicitly support timely PC access for people with HFpEF.

### Limitations

Only the lead HF clinicians across Northern Ireland were recruited for this study, which may have limited the depth of responses. We also relied on the HF lead to pass the survey link to the relevant person(s) in their service to provide additional information where needed. Therefore, we cannot rule out the possibility that someone with less oversight of the service may have completed the survey.

## Conclusions

This study highlighted key gaps in PC provision. Inconsistent completion of ACPs was one gap highlighted, where those with HF are not consistently being given the opportunity to complete ACPs. Holistic PC needs assessment was also lacking across the area. Another identified gap was the lack of locally agreed eligibility criteria for referral to specialist PC, and furthermore, specialist PC physicians are not being routinely included in multidisciplinary meetings. However, it is heartening to know that the majority of clinicians who took part in this study saw the value in PC services. Findings suggest the need for clearer materials for HF clinicians, such as systematic tools for holistic PC needs assessment, implementation of ACPs, and standardized eligibility criteria for referrals. It would also be advised that assessment for PC needs is not left to the end-of-life stage, and that those with HFpEF are given the opportunity to avail of PC comparably to those with HFrEF. With the implementation of these recommendations, it may yet be feasible for the integration of PC services into HF services to be realized.

## Acknowledgments

The authors would like to thank our research advisory board for their invaluable support for this project. This included: Linda Cooper, carer representative; Nicole McKelvie, Heart Failure Warriors NI; Edith Donnelly, Southern Trust; Liam Duggan, British Heart Foundation; Darren French, Northern Ireland Chest Heart & Stroke; Diane Walker, Palliative Care in Partnership; and Christine Irvine, Marie Curie.

## Supplementary Material
