# Supplementary material for: Palliative Care and Heart Failure Services: Mapping National Data to Inform Policy and Service Provision
Source: J Cardiovasc Nurs. 2026 Feb 17;41(6):525–33. doi: 10.1097/JCN.0000000000001312 (PMC13649280; doi:10.1097/JCN.0000000000001312)
Supplement: Supplementary file 1 [file jcn-41-0525-s001.pdf]

INTRODUCTION

Mapping exercise: supportive, palliative and end-of-life care services for people with heart failure.

We would like to improve the availability of palliative care services to people with symptomatic, advanced heart failure. Even now, too few people with heart failure have ready access to high quality palliative and end-of-life care. We are conducting this questionnaire survey to gain an understanding of the current provision of palliative care for people with heart failure in Northern Ireland (NI).

We have approached you because you are the clinical lead for heart failure services in your Health and Social Care Trust. We ask that you answer as many questions as you possibly can, and then share the link with a member of your team who can complete the rest. This is really important for us to get a complete understanding of palliative care provision for people with heart failure within your service area.

There is, of course, no obligation to fill in the questionnaire, but we would be very grateful for the 10 to 15 minutes of your time that we think it might take you.

The results will be used to find examples of good practice and service delivery as well as gaps, with the aim of helping to support individual heart failure services to develop links to palliative care where needed.

All information and data collected will be managed confidentially, in line with General Data Protection Regulations (GDPR) 2018. Aggregated anonymised information will be shared with other stakeholders e.g., to use as a national benchmark for service development purposes. Any participant identifiable data will be removed from individual survey responses, so responses remain anonymous. However, individual Trusts will need to be identified in order to map the supportive, palliative, and end-of-life care services available for individuals with advanced heart failure across regional heart failure services, but only service level data will be published. Data will be stored securely on a Queen's University Belfast database only seen by the authorised researchers.

If you have any further questions about the survey, please contact [anon] at [anon].

By completing this survey, you are consenting to participate in this study

TO BE COMPLETED BY CLINICAL SERVICE LEAD WITH SUPPORT FROM RELEVANT TEAM MEMBERS (if you are unable to complete any questions, please send the survey link to the relevant person in your team who can complete them)

SECTION 1 – QUESTIONS ABOUT SERVICES FOR PEOPLE WITH HEART FAILURE WITHIN YOUR TRUST AREA (Statutory and non-statutory)

Questions about services for people with heart failure within your Trust area (statutory and non-statutory):

1. Name of health care trust: *FREE TEXT BOX*

2. Trust name: *DROP DOWN BOX of all Trusts.*
    a. *Northern*
    b. *Western*
    c. *Southern*
    d. *Belfast*
    e. *South eastern*

3. Profession of Clinical Lead: *please tick one DROP DOWN MENU*
    a. *Cardiologist*
    b. *Cardiologist with specialist Heart Failure interest*
    c. *GP with a Special Interest*
    d. *Heart Failure Nurse*
    e. *Allied Health Practitioner*
    f. *Advanced Nurse Practitioner*
    g. *Other (please specify) FREE TEXT BOX*

4. Profession of person completing survey (if different to above): *please tick one DROP DOWN MENU*
    a. *Cardiologist*
    b. *Cardiologist with specialist Heart Failure Interest*
    c. *GP with a Special Interest*
    d. *Heart Failure Nurse*
    e. *Allied Health Practitioner*
    f. *Advanced Nurse Practitioner*
    g. *Other (please specify) FREE TEXT BOX*

5. Organisation type: *please tick one DROP DOWN MENU*
    a. *Trust Community Service*

   b. *Trust Hospital Service*
   c. *Other (describe)*

6. How would you describe your locality, in the main? *Please tick one. DROP DOWN MENU*
   a. *Urban*
   b. *Rural (sparsely populated relatively isolated)*
   c. *Mixed*

TO BE COMPLETED BY CLINICAL SERVICE LEAD WITH SUPPORT FROM RELEVANT TEAM MEMBERS (if you are unable to complete any questions, please send the survey link to the relevant person in your team who can complete them)

Section 2 – QUESTIONS ABOUT ACCESS TO PALLIATIVE CARE FOR PEOPLE WITH HEART FAILURE (please answer the following questions with reference to the services provided AT YOUR TRUST)

1. **How does your service identify patients with unmet supportive and palliative care needs?** *Tick all responses that apply. DROP DOWN MENU*
   a. *Clinician opinion in response to clinical presentation by patients in out-patients, in-patient ward rounds, primary/community care contact, concerns raised by other team members etc.*
   b. *Completion of a holistic (physical, psychological, social and occupational, carer and spiritual) assessment of unmet supportive and palliative care needs*
   c. *Regular Heart Failure Multidisciplinary Team Meeting*
   d. *Other*
   e. *Don't know*

Please add to FREE TEXT for further description if necessary *FREE TEXT BOX*

If YES to 1b, continue.
If 1a/1c/1d, proceed to Q2.

With regards to the HOLISTIC ASSESSMENT, please answer the following questions:

1.1. Does your service conduct holistic assessment … *Tick one response. DROP DOWN MENU*
   a. *Routinely?*
   b. *Occasionally?*

1.2. Is a systematic tool/template used to guide this assessment (for example, the Needs Assessment Tool-HF; What matters when it matters; Integrated Palliative Outcome Scale [IPOS])? *Tick one response. DROP DOWN MENU*
   a. *Yes (please tell us what tool is used) FREE TEXT BOX*
   b. *No*
   c. *Don't Know*

1.3. Who conducts the assessment? *Tick all responses that apply. DROP DOWN MENU*
   a. *Cardiologist*

b. *Cardiologist with specialist Heart Failure Interest*
c. *Palliative Physician*
d. *Geriatric Physician*
e. *Physician from another medical specialty*
f. *GP with a Special Interest*
g. *GP*
h. *Heart Failure Nurse*
i. *Allied Health Practitioner*
j. *Advanced Nurse Practitioner*
k. *Palliative Care Nurse Specialist*
l. *District Nurse*
m. *Other (please specify) FREE TEXT BOX*

1.4. Does the system for assessment for unmet palliative care needs above apply equally to patients with Heart Failure with reduced ejection fraction (HFrEF) and HF with preserved ejection fraction (HFpEF)?

a. *Yes,*
b. *No (please tell us in what way) FREE TEXT BOX*
c. *Don't Know*

Please add to FREE TEXT for further description if necessary *FREE TEXT BOX*

2 Does your service refer to specialist palliative care services (services for whom palliative care is their core business)? *Tick one response. DROP DOWN MENU*

a. *Yes*
b. *No*
c. *Don't Know*

If YES to 2, continue,
If NO/DON'T KNOW to 2, proceed to Q3.

2.1. What specialist palliative care services do you have access to (over and above GP and district nursing team)? *Tick all responses that apply. DROP DOWN MENU*

a. *Hospital palliative care team*
b. *Community palliative care clinical nurse specialist*
c. *Specialist palliative medicine outpatient clinic*
d. *Marie Curie Hospice Care at Home*
e. *Trust palliative care AHPs e.g., OT, physio*
f. *Other*

Please add to FREE TEXT for further description if necessary *FREE TEXT BOX*

2.2. Do you have access to a specialist palliative medicine consultant? *Tick one response. DROP DOWN MENU*

a. *Yes*
b. *No*
c. *Don't Know*

2.3. Do you have locally agreed eligibility criteria for specialist palliative care referral? *Tick one response. DROP DOWN MENU*

a. *Yes*
b. *No*
c. *Don't Know*

If YES to 2.3, continue,
If NO/DON'T KNOW to 2.3, proceed to Q3.

2.4. Please state what they are. *Tick all responses that apply. DROP DOWN MENU*

a. *We've got referral criteria, but I don't know what they are*
b. *Difficult to manage symptom(s)*
c. *Clinician thinks palliative care services are needed*
d. *NYHA III/IV*
e. *Expected to die in next year*
f. *Progressive renal dysfunction*
g. *Difficult persistent symptomatic hypotension not responding to regular treatments*
h. *No further cardiac treatment possible*
i. *Other*

Please add to FREE TEXT for further description if necessary *FREE TEXT BOX*

3. Does your service include completion of advance care plans? *Tick one response. DROP DOWN MENU BOTH*

a. *Yes*
b. *No*
c. *Don't Know*

If YES to 3, continue,
If NO/DON'T KNOW to 3, proceed to Q4.

3.1. Are advance care plans completed… *Tick one response. DROP DOWN MENU*

a. *Routinely?*

b. *Occasionally?*

Please add to FREE TEXT for further description if necessary *FREE TEXT BOX*

3.2. Who completes the care plan with the patient? *Tick all responses that apply. DROP DOWN MENU*

a. *District Nurses*
b. *Palliative Care Nurse Specialist*
c. *Advanced Nurse Practitioner*
d. *Allied Health Practitioner*
e. *Heart Failure Nurse*
f. *Cardiologist*
g. *Cardiologist with specialist Heart Failure interest*
h. *Palliative Physician*
i. *Geriatric Physician*
j. *Physician from another medical specialty*
k. *GP with a Special Interest*
l. *GP*
m. *Other (please specify) FREE TEXT BOX)*

Please add to FREE TEXT for further description if necessary *FREE TEXT BOX*

3.3. Who has access to a patient's advance care plan? *Tick all responses that apply. DROP DOWN MENU:*

a. *Don't know*
b. *Patient has a copy*
c. *All hospital staff (including emergency department) via the electronic clinical record*
d. *All hospital staff (including emergency department) through "paper" clinical records*
e. *Hospice, if involved in care*
f. *GP*
g. *Out-of-hours service*
h. *Ambulance service*
i. *Other*

Please add to FREE TEXT for further description if necessary *FREE TEXT BOX*

3.4. Is a systematic tool/template used to guide this assessment (**for example: advance care plans**)? *Tick one response. DROP DOWN MENU*

a. *Yes (please tell us what tool is used) FREE TEXT BOX*
b. *No*
c. *Don't Know*

Please add to FREE TEXT for further description if necessary *FREE TEXT BOX*

4. Does your service have a <u>Heart Failure</u> Multi-Disciplinary Team meeting? *Tick one response. DROP DOWN MENU BOTH*

a. *Yes*
b. *No*
c. *Don't Know*

If YES to 4, continue,
If NO/DON'T KNOW to 4, proceed to Q5.

4.1. Does a specialist palliative care clinician attend? *Tick one response. DROP DOWN MENU*

a. Yes, usually
b. Yes, sometimes
c. No
d. Don't Know

4.2. Do you have access to joint cardiology/palliative review of patients? Tick all that apply. DROP DOWN MENU

a. Home Visit
b. Out-patient/GP practice consultation
c. In-patient review
d. Don't know

4.3. How do you communicate plans to address unmet supportive and palliative needs with primary care? *Tick all responses that apply. DROP DOWN MENU*

a. Via Heart Failure Nurse Specialist
b. Via Palliative Care Nurse Specialist
c. Letter/email
d. Electronic Care records
e. Other (please state)

Please add to FREE TEXT for further description if necessary *FREE TEXT BOX*

5. Have your heart failure health and social care professionals engaged in any of the following palliative care training options? *Tick one response. DROP DOWN MENU*

a. Princess Alice Essential Certificate in Palliative Care
d. Placement with specialist palliative care team (hospice or hospital)
e. Palliative care in-person teaching facilitated by palliative care team

Please add to FREE TEXT for further description if necessary *FREE TEXT BOX*

6. Do you provide any heart failure teaching to palliative care clinicians? *Tick one response. DROP DOWN MENU*

a. Yes
b. No
c. Don't Know

Please add to FREE TEXT for further description if necessary *FREE TEXT BOX*

7. Please rate the following palliative care service provision in terms of importance to patients with heart failure.

a. Patient identification

Not at all important	Slightly important	Somewhat important	Moderately important	Very important

b. Comprehensive holistic assessment

Not at all important	Slightly important	Somewhat important	Moderately important	Very important

c. Access to specialist palliative care

Not at all important	Slightly important	Somewhat important	Moderately important	Very important

d. Anticipatory care planning

Not at all important	Slightly important	Somewhat important	Moderately important	Very important

e. Interdisciplinary working

Not at all important	Slightly important	Somewhat important	Moderately important	Very important

f. Training/education in palliative care for Heart Failure clinicians

Not at all important	Slightly important	Somewhat important	Moderately important	Very important

8. Does your service have palliative care and end-of-life care included in current service specifications and current business plans for service improvement? *Tick one response: DROP DOWN MENU.*

a. Yes
b. No
c. Don't Know

Please add to FREE TEXT for further description if necessary *FREE TEXT BOX*

9. Are you happy for us to use anonymised direct quotes from any free text provided, for the purpose of the executive report or publications? *Tick one response. DROP DOWN MENU*

a. Yes
b. No

Thank you for taking the time to complete this survey. Your responses are confidential and will only be used for the purposes of this study.
