# Supplementary material for: Palliative Care and Heart Failure Services: Mapping National Data to Inform Policy and Service Provision
Source: J Cardiovasc Nurs. 2026 Feb 17;41(6):525–33. doi: 10.1097/JCN.0000000000001312 (PMC13649280; doi:10.1097/JCN.0000000000001312)
Supplement: Supplementary file 2 [file jcn-41-0525-s002.pdf]

Supplementary file 2.

Table aligned to figure 1.

**Who conducts the assessment? Multiple answers allowed.**	**Trust 1**	**Trust 2**	**Trust 3**	**Trust 4**	**Trust 5**
Cardiologist		✓ (n=1)			N/A
Cardiologist with specialist Heart Failure Interest		✓ (n=1)	✓ (n=1)	✓ (n=1)	N/A
Palliative Physician					N/A
Geriatric Physician					N/A
Physician from another medical specialty					N/A
General Practitioner with a Special Interest					N/A
General Practitioner					N/A
Heart Failure Nurse	✓ (n=1)	✓ (n=1)	✓ (n=1)	✓ (n=1)	N/A
Allied Health Practitioner					N/A
Advanced Nurse Practitioner	✓ (n=1)		✓ (n=1)		N/A
Palliative Care Nurse Specialist					N/A
District Nurse					N/A

***Only the respondents who answered yes to completion of holistic assessment answered rest of question 1, and the others moved on to question 2.**

Table aligned to figure 4.

**Who completes the care plan with the patient?**	**Trust 1**	**Trust 2**	**Trust 3**	**Trust 4**	**Trust 5**
District Nurses		✓ (n=1)		N/A	
Palliative Care Nurse Specialist		✓ (n=1)		N/A	
Advanced Nurse Practitioner		✓ (n=1)		N/A	✓ (n=1)
Allied Health Practitioner				N/A	
Heart Failure Nurse				N/A	
Cardiologist				N/A	
Cardiologist with specialist Heart Failure interest				N/A	
Palliative Physician	✓ (n=1)	✓ (n=1)	✓ (n=1)	N/A	✓ (n=1)
Geriatric Physician				N/A	
Physician from another medical specialty	✓ (n=1)			N/A	
GP with a Special Interest		✓ (n=1)		N/A	✓ (n=1)
GP				N/A	

Table aligned to figure 5.

How do you communicate plans to address unmet supportive and palliative needs with primary care?	Trust 1	Trust 2	Trust 3	Trust 4	Trust 5
Via Heart Failure Nurse Specialist	N/A	✓ (n=3)	✓ (n=1)		✓ (n=1)
Via Palliative Care Nurse Specialist	N/A	✓ (n=3)		✓ (n=1)	✓ (n=1)
Letter/email	N/A	✓ (n=2)		✓ (n=3)	
Electronic Care records	N/A	✓ (n=3)			✓ (n=1)
Other				✓ (n=1)	

Tabel aligning with figure 6.

Have your heart failure health and social care professionals engaged in any of the following palliative care training options?	Trust 1	Trust 2	Trust 3*	Trust 4	Trust 5
Princess Alice Essential Certificate in Palliative Care		✓ (n=1)			
Placement with specialist palliative care team (hospice or hospital)	✓ (n=1)		✓ (n=1)	✓ (n=1)	
Palliative care in-person teaching facilitated by palliative care team		✓ (n=2)		✓ (n=2)	✓ (n=1)

*One respondent did not answer.
