# Supplementary material for: Palliative Care and Heart Failure Services: Mapping National Data to Inform Policy and Service Provision
Source: J Cardiovasc Nurs. 2026 Feb 17;41(6):525–33. doi: 10.1097/JCN.0000000000001312 (PMC13649280; doi:10.1097/JCN.0000000000001312)
Supplement: Supplementary file 3 [file jcn-41-0525-s003.pdf]

Supplementary file 2

Supplementary table: Please rate the following palliative care service provision in terms of importance to patients with heart failure.

**Patient identification**	**Not at all important**	**Slightly important**	**Somewhat important**	**Moderately important**	**Very important**
Trust 1					✓(n=1)
Trust 2					✓(n=3)
Trust 3			✓(n=1)		✓(n=1)
Trust 4					✓(n=3)
Trust 5					✓(n=1)
**Comprehensive holistic assessment**	**Not at all important**	**Slightly important**	**Somewhat important**	**Moderately important**	**Very important**
Trust 1					✓(n=1)
Trust 2					✓(n=3)
Trust 3			✓(n=1)		✓(n=1)
Trust 4			✓(n=1)		✓(n=2)
Trust 5					✓(n=1)
**Access to specialist palliative care**	**Not at all important**	**Slightly important**	**Somewhat important**	**Moderately important**	**Very important**
Trust 1				✓(n=1)	
Trust 2					✓(n=3)
Trust 3			✓(n=1)		✓(n=1)
Trust 4			✓(n=1)		✓(n=2)
Trust 5					✓(n=1)
**Anticipatory care planning**	**Not at all important**	**Slightly important**	**Somewhat important**	**Moderately important**	**Very important**
Trust 1					✓(n=1)
Trust 2					✓(n=3)
Trust 3			✓(n=1)	✓(n=1)	
Trust 4		✓(n=1)			✓(n=2)
Trust 5					✓(n=1)
**Interdisciplinary working**	**Not at all important**	**Slightly important**	**Somewhat important**	**Moderately important**	**Very important**
Trust 1					✓(n=1)
Trust 2					✓(n=3)
Trust 3			✓(n=1)		✓(n=1)
Trust 4			✓(n=1)		✓(n=2)

Trust 5					✓(n=1)
**Training/education in palliative care for Heart Failure clinicians**	**Not at all important**	**Slightly important**	**Somewhat important**	**Moderately important**	**Very important**
Trust 1					✓(n=1)
Trust 2					✓(n=3)
Trust 3			✓(n=1)		✓(n=1)
Trust 4				✓(n=1)	✓(n=2)
Trust 5					✓(n=1)
